# Impact of USMLE Step 1 transition to pass/fail scoring system on medical students’ anxiety, sleep quality, and burnout

**DOI:** 10.1007/s11845-024-03738-x

**Published:** 2024-07-02

**Authors:** Lana Al Doori, Peter G. Zaki, Vivek Joshi

**Affiliations:** 1 Drexel University College of Medicine at Tower Health, Drexel University, West Reading, Wyomissing, PA 19610 USA; 2 Department of Biochemistry, Kasturba Medical College Manipal, Manipal Academy of Higher Education, Manipal, Karnataka India

**Keywords:** Anxiety, Burnout, Medical students, Sleep quality, USMLE

## Abstract

**Introduction:**

Originally designed to evaluate the application of foundational scientific knowledge in clinical contexts, the United States Medical License Exam (USMLE) Step 1 evolved into a comparative tool for assessing candidates with similar educational foundations. This transition heightened the pressure on medical students to excel in the exam. In response, collaborative efforts involving the National Board of Medical Examiners prompted a change from reporting scores to a pass/fail system. The true impact of this shift remains insufficiently explored. This study aims to assess the emotional toll — encompassing burnout, anxiety, depression, and sleep quality — experienced prior to taking the Step 1 exam. Additionally, it aims to uncover potential gender-based disparities in perceived anxiety and depression.

**Methods:**

The study encompasses the entirety of third-year medical students at Drexel University College of Medicine, who were invited to participate in a comprehensive survey. Drawing from retrospection, the survey relies on self-reported data regarding anxiety, depression, sleep quality, and burnout. Data compilation was anonymized and executed via Qualtrics platform.

**Results:**

A total of 102 medical students completed the survey, with a 97% pass rate for the USMLE Step 1. Despite their excellent performance, 75% of students reported inadequate sleep quality, and 68% exhibited mild to moderate anxiety levels. Among them, a higher percentage of females (83%) experienced anxiety compared to their male counterparts (50%). Furthermore, 66% of students felt that their commitment to education exceeded what was reasonable for their well-being.

**Conclusions:**

The transition from traditional scoring to a pass/fail system was ostensibly intended to enhance the mental well-being of medical students. Nevertheless, our findings underscore that students continue to endure heightened levels of stress, anxiety, and burnout during the pivotal month leading up to the Step 1 examination.

## Introduction

The conventional structure of medical education involves a preliminary two-year phase focused on fundamental scientific principles, preceding the clinical phase. Prior to starting third-year clerkships, many medical schools require students to pass the first of three medical licensing exams: the United States Medical Licensing Exam (USMLE) Step 1. Introduced in 1992, USMLE Step 1 places emphasis on applying foundational basic science knowledge to clinical practice [1–3].

Historically, the National Board of Medical Examiners (NBME) employed a numerical scoring system to provide students with performance feedback relative to passing thresholds. Gradually, residency program directors began to use Step 1 scores as a core criterion for comparing applicants with similar educational backgrounds. The imbalance between the number of residency applicants and the available positions, particularly from 2009 to 2019 when there were only 0.8 positions per applicant, has been striking. An astonishing 94% of program directors considered Step 1 scores as the most critical factor in determining interview invitations. Consequently, the average USMLE Step 1 score has exhibited a consistent upward trend, with a mean score of 200 in 1993 equating to only the 8th percentile in 2018 [4–6].

Unfortunately, the high stakes associated with the exam unintentionally harmed medical students. A growing number of students are choosing to defer their medical school curricula and turn to commercial resources to prepare for the test [6]. Studies indicated that 70% of students experienced anxiety and stress due to the misalignment between the medical school curriculum and USMLE Step 1 content [11]. Recognizing the high stakes, companies capitalized on students’ stress and anxiety by creating resources with concise, clear designs that emphasized the most “high yield” topics. Furthermore, the plethora of available resources and the financial strain of purchasing essential content mastery assessments, such as the NBME practice tests, only serve to compound student’s distress [6–8].

In contrast, pre-med students typically exhibit healthier coping mechanisms compared to their counterparts already enrolled in medical school. However, the well-being of medical students often experiences a sharp decline upon their entry into the rigorous academic environment of medical school. Most medical schools allocate 4 to 6 weeks’ time (dedicated) for students to focus solely on studying for the Step 1 exam. During this period, students endure extended hours of intense study, which can lead to feelings of isolation, stress, exhaustion, and burnout [9, 10]. Recognizing this mental health crisis, the NBME and Federation of State Medical Boards (FSMB) decided to transition the Step 1 score reporting system to pass/fail [4]. However, the effectiveness of this transition remains inadequately explored.

This study aims to comprehensively assess the emotional toll manifested through burnout, anxiety, depression, and sleep quality leading up to the Step 1 exam. Additionally, it endeavors to unveil any potential gender-based disparities in perceived stress, anxiety, and burnout.

## Methods

A total of 102 third-year medical students from Drexel College of Medicine were enrolled. A non-probability sampling method was used to send emails to all third medical students who have written the USMLE Step 1 exam.

This study depended on retrospective self-reported data on self-anxiety scale, patient health questionnaire, sleep quality index, rating of burnout level, and coping mechanisms utilized. This data was collected via Qualtrics. All the survey questionnaires were submitted anonymously; student names, emails, and identifying information were not required while filling the survey questionnaire.

### Data collection

The sleep quality index scale contains seven components: subjective sleep quality, sleep latency, sleep duration, habitual sleep efficiency, sleep disturbances, use of sleep medication, and daytime dysfunction. There are 8 questions in this questionnaire, with a total score ranging from 0 to 21. A higher score indicated worse sleep quality. Using a global score > 5 and ≤ 5, patients are divided into a poor sleeper’s group and a good sleeper’s group.

Self-rating anxiety scale was determined by a crude scale score, equal to the sum of each item score. The total score was then classified into minimal anxiety — 0 to 8 point, mild anxiety — 9 to 16 points, moderate anxiety — 17 to 24 points, high anxiety (Warning Level) — 25 to 32 points, extreme anxiety (Warning Level) — 33 to 40 points. This questionnaire has 7 items.

The Patient Health Questionnaire depression scale (PHQ) is established as a valid diagnostic and severity measure for depressive disorders in large clinical studies. The scores range from 0 to 24. A higher score indicates a higher level of depression.

Burnout Assessment Tool questionnaire is divided into three domains including emotional exhaustion (EE), disengagement from work or depersonalization (DP), and personal achievement or accomplishment (PE). This inventory contains 7 items scored on a Likert scale ranging from strongly agree to strongly disagree (4-point Likert scale).

All the data will be entered into the Microsoft Excel sheet. The data is represented as mean and standard deviation. To simplify the reporting of findings of students on four points Likert scale, % agreement (strongly agree + agree) and % disagreement (strongly disagree + disagree) categories will be combined. The qualitative data derived from qualitative questionnaire is represented in terms of percentage and “p” value as significance. The statistical significance is defined as a *p*-value of < 0.05. To achieve a power of 0.80 with a medium effect size (Cohen’s *d* = 0.5) and a significance level (alpha) of 0.05, the required sample size for each group in an independent *t*-test would be approximately 64 participants.

## Results

The study involved 102 participants of both genders. Anxiety, PHQ, and quality of sleep levels are shown in Table 1.

**Table 1:** Participants’ anxiety and PHQ values

	Number	Mean	SD
Anxiety	95	17.07	9.52
PHQ	95	9.59	6.70
Quality of sleep	68	9.11	4.39

Table 2 shows the gender-wise comparison, which showed there was no significant difference in anxiety levels and PHQ values among both groups. However, the PHQ scores were positively correlated with anxiety levels in both male and female subjects as depicted in Table 3.

**Table 2:** Comparison of gender-wise anxiety and PHQ values

	Male (41)	SD	Female (52)	SD	*p*-value
Anxiety levels	16.00	10.84	17.88	8.53	.617
PHQ levels	9.47	7.89	9.68	5.81	.890

**Table 3:** Correlation of various parameters in male and female participants

Correlations in males			
		Anxiety	PHQ
Anxiety	Pearson correlation	1	.936**
	Sig. (2-tailed)		** < .001**
PHQ	Pearson correlation	.936**	1
	Sig. (2-tailed)	** < .001**	
Correlations females			
		**Anxiety**	**PHQ**
Anxiety	Pearson correlation	1	.789**
	Sig. (2-tailed)		** < .001**
PHQ	Pearson correlation	.789**	1
	Sig. (2-tailed)	< .001	

^**^Correlation is significant at the 0.01 level (2-tailed)

Table 4 shows the values based on anxiety levels; the study showed that higher anxiety was associated with higher scores of PHQ which was statistically significant. The scores of participants based on PHQ scores also showed that higher PHQ scores were also associated with high anxiety levels in the study group which showed statistically significant as shown in Table 4.

**Table 4:** Various values based on levels of anxiety in participants

	Normal anxiety levels (54)	SD	High anxiety levels (20)	SD	*p*-value
PHQ levels	6.97	4.64	18.50	4.65	** < .001**
	**Normal PHQ (54)**	**SD**	**High PHQ (20)**	**SD**	***p*-value**
Anxiety levels	13.47	6.69	29.30	7.33	** < .001**

Based on the type of study (group vs. solo), there were no significant changes in anxiety and PHQ scores. But on correlation, both groups showed anxiety and PHQ scores positively correlated as represented in Tables 5 and 6.

**Table 5:** Various values based on type of study (group vs. solo) in participants

	Solo (53)	SD	Group (31)	SD	*p*-value
Anxiety levels	17.39	8.97	16.09	11.44	.700
PHQ levels	9.91	6.67	8.64	7.03	.262

**Table 6:** Correlations of participants with type of study

Solo study		Anxiety	PHQ
Anxiety	Pearson correlation	1	.878**
	Sig. (2-tailed)		** < .001**
PHQ	Pearson correlation	.878**	1
	Sig. (2-tailed)	< .001	
Group study		**Anxiety**	**PHQ**
Anxiety	Pearson correlation	1	.856**
	Sig. (2-tailed)		** < .001**
PHQ	Pearson correlation	.856**	1
	Sig. (2-tailed)	< .001	

Table 7 shows the percentage of students experiencing low and high quality of sleep. Table 8 indicates student agreement to two different statements regarding prioritization of well-being versus educational investment.

**Table 7:** Quality of sleep

	Percent of students
Poor quality of sleep	73.91
Good quality of sleep	26.09
	Score
Mean (SD)	9.11 (4.39)

**Table 8:** Percent of students agree with statements regarding educational investment

“I overlook and neglect my personal life when I pursue important achievement and educational demands”	Percent of students
Agree	83.83
Somewhat Disagree	16.17
“I think the dedication I invest in my education is more than what it should be relative to my health”	
Agree	66.18
Disagree	33.82

## Discussion

### Impact of pass/fail scoring on stress and career path

The change in USMLE Step 1 score reporting to pass/fail was intended to alleviate students’ anxiety, despite this transition, our study results reveal that students continue to experience elevated anxiety, depression, and poor sleep quality as depicted in Table 1. Step 1 remains an essential milestone in medical education, as it serves as both a graduation requirement and a prerequisite for commencing third-year rotations. Medical students are granted up to six attempts to pass this examination, and while it is forgiving, failing Step 1 on the first attempt can significantly affect students’ prospects, particularly when applying for residency programs. This impact is especially pronounced for students applying to historically competitive specialties such as ENT, plastic surgery, neurosurgery, orthopedics, and dermatology. Notably, a survey of 1200 residency program directors revealed that a third of them would never consider applicants who have failed Step 1 on their initial attempt. Thus, certain career paths are potentially closed off for students applying to these competitive specialties [8–10].

### Comparative analysis: pass/fail vs. scored system

Our findings indicate that students continue to struggle with stress, anxiety, and burnout even under the pass/fail scoring system. To provide context and perspective, we turn to a comparative analysis between students who underwent Step 1 under the new pass/fail system and those who faced the traditional scored examination:

A study conducted by Baniadam et al. offers insights into the overall stress levels between these two cohorts. Interestingly, the study suggests that students in both groups exhibited similar overall stress levels. However, the dynamics of stress differed at various stages of their medical education. Furthermore, Baniadam et al. showed that during early and mid-second year, students under the scored system reported heightened stress levels compared to their pass/fail counterparts. This discrepancy could be attributed to the pressure associated with preparing for a high-stake scored examination during this period. Nevertheless, stress levels among the pass/fail cohort began to converge with those of the scored cohort during the dedicated study period for Step 1. Additionally, the pass/fail students experienced heightened stress in relation to Step 2, pre-clerkship grades, and clerkships, ultimately reaching the same stress levels as their counterparts who took Step 1 as a scored exam [2].

This comparison highlights the complex interplay of stress factors within medical education and underscores that transitioning to a pass/fail system does not eliminate the overall stress experienced by medical students.

### Anxiety and cognitive performance

Our survey results reveal that 35% of students experienced moderate to high levels of anxiety during the period leading up to the Step 1 exam. Anxiety manifests in two key aspects: cognitive and physiological. Physiologically, it leads to nervousness, elevated heart rate, and sweating — a response driven by an overstimulated sympathetic nervous system. However, research indicates a lack of a direct relationship between these physiological changes and academic achievement. Conversely, anxiety’s cognitive component, marked by negative thoughts of failure, self-doubt, and unpreparedness, significantly hampers intellectual task performance. This cognitive aspect of anxiety diverts cognitive resources away from the task at hand, impacting working memory and the ability to concentrate, ultimately hindering students from reaching their full potential [11–13].

### Sleep deprivation and cognitive function

Our data unveils that a striking 74% of students experienced poor sleep quality during the period leading up to the Step 1 exam. This prevalence of sleep disturbances is notably higher among medical students compared to the general population. The significance of high-quality sleep cannot be overstated, as it plays a pivotal role in long-term memory, neurocognitive function, mental well-being, and physical health. Conversely, inadequate sleep predisposes individuals to anxiety and depression, diminished quality of life scores, and a deteriorated perception of the educational environment [14].

The consequences of sleep deprivation on neurocognition are profound, affecting executive attention, working memory, and higher cognitive functions. Neurophysiological and metabolic studies emphasize the heightened susceptibility of executive function impairment mediated by the prefrontal cortex to sleep deprivation. Furthermore, sleep deprivation elevates the risk of human-error-related accidents. The two-process model of sleep, encompassing the sleep homeostatic process (process S) and the circadian process (process C), explains the temporal patterns of sleep and wakefulness. During sleep deprivation, process S takes precedence, impairing neurocognitive function even during peak wakefulness. The functional level of sleep-deprived individuals falls to an estimated 9th percentile compared to non-sleep-deprived counterparts. Notably, partial sleep deprivation (less than 7 h in a 24-h period) appears to impact mood and cognition more severely than total sleep deprivation (greater than 45 h without sleep). Partially sleep-deprived individuals performed two standard deviations below non-sleep-deprived controls. Sleep deprivation affects a range of cognitive functions, including delayed response time, decline in short-term recall and working memory performance, reduced acquisition of cognitive tasks, performance deterioration with increasing duration, and disruption of mental tasks requiring divergent thinking. While sleep deprivation has limited effect on novel logic-based tasks, it substantially impairs performance in tasks demanding creative thinking, which heavily relies on executive function, working memory, and attention systems. Neurocognitive deficits interfere with working memory tasks, making it difficult to determine problem scope, maintain temporal memory, sustain focus, engage in flexible thinking, control impulsivity, and exhibit performance insight. It also impedes behavioral adaptations based on new information. Consequently, sleep deprivation significantly impairs executive functions and working memory associated with the prefrontal cortex [7, 8].

Beyond its impact on the prefrontal cortex, sleep deprivation adversely affects cognitive function, particularly hippocampal function, impairing synaptic plasticity and transmission. Memory consolidation, the process of storing information in the brain following initial acquisition, is notably influenced by sleep. In the immediate hours following the acquisitions of new information, synaptic consolidation occurs, leading to the formation of new synaptic connections and the restructuring of existing ones. Sleep deprivation disrupts cAMP-dependent hippocampal CA1 neuron synaptic plasticity associated with long-term potentiation and memory consolidation. Just a few hours of sleep deprivation results in the loss of dendritic spines in CA1 neurons [3]. Sleep promotes dendritic spine formation in neurons activated during learning, with a 3-h sleep recovery effectively restoring spine numbers in CA1 neurons and mitigating long-term potentiation deficits caused by sleep deprivation [9].

The prevalence of poor sleep quality (74%) among students, notably impacts cognitive functions like attention, memory, and mental well-being. Sleep deprivation, especially before exams, impairs learning, memory consolidation, and overall cognitive abilities. These findings highlight the critical importance of good quality of sleep for academic performance and mental health in students. The universities should take appropriate measures to mitigate the effects on mental health and student well-being by offering workshops and seminars on stress management techniques, mindfulness, and meditation to help students develop healthy coping mechanisms. The universities should also provide education on the importance of sleep and strategies to improve sleep hygiene, encourage students to take short restorative breaks during the study sessions. The other recommendation based on our study is to incorporate mental health education into medical curriculum to raise awareness about mental health issues, reduce stigma, and teach students resilience skills.

### Burnout and suicidal ideation

Of the students surveyed 83% agreed with the statement “I overlook and neglect my personal life when I pursue important achievement and educational demands.” Additionally, 66% agreed that “the dedication I invest in my education is more than what it should be relative to my health.” Both statements are indications of burnout. Evidence suggests that there is an association between medical student burnout and suicidal ideation. Studies show that students experiencing burnout are three times more likely to have considered suicide in the past. This association persisted even when controlling for depression within the same population. Burnout was also linked to serious thoughts of dropping out of medical school [12, 15, 16].

## Conclusion

Our study’s results indicate that students are still facing moderate to high levels of anxiety and mild levels of depression, which can impede their cognitive function. Additionally, a substantial number of students are struggling with poor sleep quality. What’s particularly concerning is that partial sleep deprivation has a more severe impact on cognition than total sleep deprivation. Moreover, sleep deprivation predisposes students to the development of depression, anxiety, and burnout. These emotional challenges have the potential to adversely affect various cognitive functions, including working memory, memory consolidation, and decision-making abilities. Despite the shift from numerical scoring to a pass/fail system, the reduction in student anxiety remains elusive.

## Limitations

To gain a more comprehensive understanding of the factors influencing the mental health of medical students, further research is imperative. Our study had some limitations, primarily related to data collection at a single point in time rather than over the entire academic year. Additional constraints include the absence of data on various factors impacting the well-being of medical students, such as access to resources, nutritious food, exercise, and socioeconomic status, all of which can contribute to stress and anxiety.

## Data Availability

The data generated during the research and analysis are not available publicly but are available from the corresponding author on a reasonable request.
